# Longitudinal changes in plasma biomarkers of immune activation, neuronal inflammation and injury in persons with HIV initiating ART

**DOI:** 10.1111/hiv.70236

**Published:** 2026-04-16

**Authors:** Merle Henderson, Peter Dutey‐Magni, Carolina Herrera, Wolfgang Stöhr, Alejandro Arenas‐Pinto, Owen Swann, Amanda Heslegrave, Henrik Zetterberg, John Tregoning, Sarah Fidler, François Raffi, Andrea Calcagno, Ab Babiker, Alan Winston

**Affiliations:** ^1^ Department of Infectious Disease Faculty of Medicine, Imperial College London London UK; ^2^ NIHR Imperial College London Biomedical Research Centre London UK; ^3^ Jefferiss Wing, St. Mary's Hospital, Imperial College Healthcare NHS Trust London UK; ^4^ MRC Clinical Trials Unit University College London London UK; ^5^ CONRAD, Macon & Joan Brock Virginia Health Sciences at Old Dominion University Norfolk Virginia USA; ^6^ UK Dementia Research Institute, University College London London UK; ^7^ Department of Neurodegenerative Disease UCL Institute of Neurology, Queen Square London UK; ^8^ Department of Psychiatry and Neurochemistry Institute of Neuroscience and Physiology, The Sahlgrenska Academy at the University of Gothenburg Mölndal Sweden; ^9^ Clinical Neurochemistry Laboratory Sahlgrenska University Hospital Mölndal Sweden; ^10^ Hong Kong Center for Neurodegenerative Diseases, InnoHK Hong Kong China; ^11^ Wisconsin Alzheimer's Disease Research Center, University of Wisconsin School of Medicine and Public Health, University of Wisconsin‐Madison Madison Wisconsin USA; ^12^ Centre Hospitalier Universitaire Nantes France; ^13^ Department of Infectious Disease University of Turin Turin Italy

**Keywords:** antiretroviral therapy, biomarkers, central nervous system, cognitive function, neuroinflammation

## Abstract

**Background:**

Data on changes in biomarkers of brain health, and their associations with cognitive function in adults commencing either dual‐ or triple‐antiretroviral therapy (ART) are sparse.

**Methods:**

Plasma biomarkers (neurofilament light [NfL], glial fibrillary acidic protein [GFAP], sCD14, CXCL10, neopterin and IL‐6) were measured at baseline and after 96 weeks on ART in individuals randomized to darunavir/ritonavir and either tenofovir‐DF/emtricitabine (triple‐ART, *n* = 119) or raltegravir (dual‐ART, *n* = 119) in NEAT‐001/ANRS143. Regression models examined associations of baseline and week‐96 biomarker concentrations with HIV clinical parameters, composite cognitive test scores (Standardized neuropsychological test [NPZ], 7‐domains) and treatment arm.

**Results:**

In 238 individuals, median age was 38 (interquartile range [IQR] 31, 46) years, 87% male and 83% of white ethnicity. Baseline median log_10_ HIV RNA 4.73 (IQR 4.23, 5.11) copies/mL and CD4 350 (IQR 285, 412) cells/mm^3^. At baseline, higher biomarker concentrations were associated with lower CD4 (NfL, GFAP, CXCL10; *p* < 0.03), higher log_10_ HIV RNA (sCD14, neopterin, CXCL10; *p* < 0.02) and longer known duration of HIV (sCD14; *p* = 0.044). At week‐96, 94% had plasma HIV <50 copies/mL, and a decline in biomarker concentrations was observed: GFAP −14.4%, sCD14 −6.8%, neopterin −47.4%, CXCL10 −58.8%, IL‐6 −29.5% (all *p* < 0.001) and NfL −4.4% (*p* = 0.075). NPZ improved by 0.21 mean points. Change in GFAP, CXCL10, sCD14, neopterin and NfL was negatively associated with change in CD4 (all *p* ≤ 0.002) but not change in NPZ (*p* > 0.05). A greater decline in neopterin concentration was observed with dual‐ (−50.2%) versus triple‐ART (−44.3%; *p* = 0.022).

**Conclusions:**

Plasma biomarkers of brain health improved following ART initiation, associated predominantly with improvements in CD4 count and partly with treatment arm.

## INTRODUCTION

Antiretroviral therapy (ART) prevents the development of severe HIV‐associated brain disorders, and the initiation of ART is associated with improvements in cognitive performance [1, 2]. Despite ART, cognitive disorders remain prevalent in persons with HIV, which may be related to both HIV‐associated factors—such as damage sustained prior to commencing ART, persistent low‐level viral replication with resultant immune activation and neuroinflammation and ART‐related toxicity, and non‐HIV‐associated factors—such as the presence of non‐infectious comorbidities and lifestyle factors which may impact cognitive health [3].

Several cerebrospinal fluid biomarkers have been associated with cognitive function in persons with HIV. Neurofilament light chain (NfL) protein is a structural component of myelinated axons, which diffuses into the cerebrospinal fluid in response to axonal injury and acts as a sensitive marker of active central nervous system (CNS) injury [4, 5]. Glial fibrillary acidic protein (GFAP) is expressed by astrocytes and may be upregulated during astrocytic activation, triggered by HIV infection. Several other monocytic/macrophage markers have also been identified, such as C‐X‐C motif chemokine ligand 10 (CXCL10) [6], neopterin [7] and soluble CD14 (sCD14) [8]. While traditionally cerebrospinal fluid biomarkers have been used to inform the diagnosis of neuronal injury, more recent studies have demonstrated a close correlation between cerebrospinal fluid and plasma concentrations of certain biomarkers, such as NfL [9, 10]. This may be of use in several settings such as, clinical practice where cerebrospinal fluid examination is not readily available, and in clinical research where repeated cerebrospinal fluid sampling may not be appropriate or practical. The dynamics of these biomarker changes over time and their association with cognitive function in treatment naïve individuals with HIV commencing different ART regimens remains relatively unexplored. Interleukin‐6 (IL‐6) is a well‐recognized proinflammatory cytokine which is predictive of disease outcomes in persons with HIV [11]. This biomarker of peripheral inflammation may be utilized as a control biomarker to determine whether any observed neuronal inflammatory responses correlate with systemic inflammation.

ART regimens have historically comprised three agents: two nucleoside reverse transcriptase inhibitors (NRTI) and a third anchor agent from a different drug class. More recently, simplified two‐drug, ‘dual’‐ART regimens, such as those containing the integrase‐strand transfer inhibitor (INSTI) dolutegravir and the NRTI lamivudine, have demonstrated non‐inferiority in maintaining plasma suppression when compared to standard three‐drug, ‘triple’‐ART regimens [12, 13, 14]. Subsequently, dual‐ART has been incorporated into several HIV treatment guidelines [15, 16, 17]. Other dual‐ART regimens may also be used in certain circumstances that are not otherwise recommended as first‐line ART, according to certain HIV treatment guidelines, such as NRTI‐sparing regimens [17]. While initial studies have demonstrated viral suppression in the cerebrospinal fluid of persons with HIV on dual‐ART [18, 19], further work on the CNS safety of such regimens is warranted.

We aimed to determine longitudinal changes in plasma soluble biomarkers of immune activation, neuronal inflammation and injury, and their associations with cognitive function in persons with chronic HIV commencing ART for the first time with dual‐versus triple‐ART.

## METHODS

### Study design and participants

The NEAT001/ANRS143 trial was a multicentre, open‐label, randomized controlled trial comparing darunavir/ritonavir 800 mg/100 mg once daily with either raltegravir (RAL) 400 mg twice daily (dual‐ART) [17] or tenofovir disoproxil/emtricitabine 245/200 mg once daily (triple‐ART), amongst persons with HIV commencing ART for the first time [14]. Randomization was performed in a 1:1 ratio, with samples and clinical data collected between August 2010 and October 2013 across 15 European countries. A cognitive sub‐study with longitudinal cognitive assessment was performed at specific sites using validated neuropsychological tests in the main language of these countries [1]. All participants had a CD4 count of less than 500 cells/mm^3^ and/or symptomatic HIV, in line with treatment guidance at the time of recruitment [20]. Further detail on the NEAT001/ANRS143 trial and cognitive sub‐study has been previously described [1, 14]. Biomarker concentrations were measured from stored plasma samples collected at baseline (W0) and week 96 (W96). All participants provided written informed consent for future use of stored samples, which had human ethics committee approval for each study site [1, 14]. The current biomarker study included baseline participant data collected as part of the NEAT001/ANRS143 main trial and cognitive sub‐study.

### Cognitive assessment

A comprehensive battery of tests at W0 and W96 assessed seven cognitive domains specifically chosen based on those reported to be most affected by HIV disease [1]. Cognitive tests included trial making test (TMT) part A and B (attention and mental flexibility), digital substitution test (psychomotor speed), backward digit span test (working memory), free and cued selective reminding test (retrieval ability and selective memory), semantic and formal fluency tests (verbal fluency) and the frontal assessment battery (frontal executive function). Assessments were standardized across study sites and performed by trained staff [1]. Raw cognitive test scores were converted to individual domain‐specific *z*‐scores using a norming process which subtracted the mean and divided by the standard deviation (SD) of the total study sample at baseline [1]. A composite cognitive score (NPZ) was calculated as an average of the seven *z*‐scores at W0 and W96 [1].

### Laboratory analyses

Plasma samples from W0 and W96 were stored at −80°C prior to biomarker analysis and underwent two freeze–thaw cycles prior to analysis. Thawed samples were analysed according to manufacturer instructions. NfL and GFAP concentrations were measured in singlet using a Single molecule array (Simoa) assay (Quanterix, Billerica, USA) on the HD‐X Simoa instrument at the UK Dementia Research Institute, University College London (UK). Samples were randomized across assay plates. Intra‐ and inter‐assay coefficient of variation (CVs) were less than 15% and 10% respectively, as determined by 27 quality controls. CXCL10 and IL‐6 concentrations were measured in duplicate using U‐plex® multiplex immunoassays (Meso Scale Diagnostics, Rockville, USA). Enzyme‐linked immunosorbent assays (ELISA) were used to determine the plasma concentrations of neopterin (Tecan) and sCD14 (Duo‐Set ELISA, Bio‐techne, Minneapolis, USA), which were also measured in duplicate. The sCD14 ELISA was a commercial kit containing manufacturer‐validated matched antibody pairs. Intra‐ and inter‐assays CVs were not measured for U‐plex® and ELISA kits, which were commercially available, validated assays.

### Statistical analysis

The statistical analysis plan prespecified a hierarchy for the study hypotheses, with the primary hypotheses being that there was a difference in absolute concentrations of NfL and GFAP between W0 and W96. All other secondary objectives were considered exploratory, including absolute change in other biomarkers between W0 and W96 and associations with both baseline characteristics and randomized treatment arm. The purpose of this study was not to compare our findings with those of other previously reported studies; rather we aimed to determine longitudinal changes in plasma biomarker concentrations in treatment naïve persons with HIV commencing ART and their associations with other clinical and laboratory characteristics.

For descriptive characteristics, previously published age‐adjusted plasma NfL thresholds based on population data were used to determine the number of individuals with elevated W0 and/or W96 NfL concentrations (7 pg/mL 5–17 years, 10 pg/mL 18–50 years, 15 pg/mL 51–60 years, 20 pg/mL 61–70 years and 35 pg/mL >70 years) [21].

Plasma biomarker concentrations were transformed using a base 10 logarithm for analysis. Change scores were computed as the absolute difference in log_10_‐transformed concentrations. All estimates were exponentiated back (10^estimate) to interpret results: arithmetic mean log_10_‐concentrations were back‐transformed into geometric means (GM) on the untransformed scale, while regression coefficients, differences in mean log_10_ concentrations and mean log_10_ change scores were back‐transformed into relative change (RC) ratios (<1 indicating a decrease, and >1 an increase).

Student's *t*‐tests examined the significance of change between W0 and W96 for each biomarker in turn. Univariable and multivariable linear regression models then evaluated the association between the biomarker value at W0 and baseline characteristics: age (centred on 40 and divided by 10, with best fitting first‐order fractional polynomial transformation), sex, CD4 count (<50, 50–199, 200–349, 350–499, ≥500 cells/mm^3^), years in education (continuous, centred on 13), duration of HIV infection (divided by 5), smoking status (never smoked, previous smoker, current smoker) and NPZ score. Multivariable linear regressions, adjusted for age and sex, evaluated the association between biomarker change scores with absolute change in the following factors, one at a time: CD4 count (log_10_‐transformed), CD8 count and NPZ score between weeks 0 and 96. Statistical significance was assessed based on comparing each model against a model including just age and sex, using *F* tests. A final set of regressions analysed the effect of change in CD4 count, CD8 count and NPZ score together in addition to age and sex. Analysis of covariance (ANCOVA) models adjusted for age, sex and baseline biomarker value compared the effect of dual‐ and triple‐ART on biomarker change between W0 and W96. Analyses were performed using R version 4.4.1 and observed a formal statistical analysis plan. Data were assumed to be missing completely at random. Statistical hypotheses were rejected at the 0.05 significance level.

## RESULTS

### Participant characteristics

Of 805 individuals enrolled in the NEAT001/ANRS143 trial (343 in the cognitive sub‐study), 238 had stored plasma samples at W0 and W96 and were included in this biomarker analysis, of which 170 had corresponding longitudinal cognitive assessments (CONSORT diagram Figure S1). Of these 238 individuals, 119 were randomized to dual‐ART and 119 to triple‐ART (Table 1) with longitudinal cognitive assessments available in 75 and 95, respectively (*p* = 0.002). The median age was 38 (IQR 31, 46) years, 87% were male and 12% of black ethnicity. Median log_10_ HIV RNA was 4.73 (IQR 4.23, 5.11) copies/mL and CD4 count 350 (IQR 285, 412) cells/mm^3^ (Table 1). Individuals enrolled in the biomarker sub‐study had a higher nadir and W0 CD4 count (nadir CD4 336 (IQR 261, 388) versus 303 (IQR 232, 367) cells/mm^3^, *p* < 0.001; W0 CD4 350 (IQR 285, 412) and 329 (IQR 244, 393) cells/mm^3^, *p* = 0.009), when compared to those not in the biomarker cohort, respectively, with no other baseline differences observed by randomization arm, gender, age, ethnicity, smoking history, education or plasma HIV RNA concentration (Table S1). No differences in baseline characteristics were observed across the biomarker cohort in those with missing NPZ scores, when compared to those without missing NPZ scores (Table S2).

**TABLE 1:** Baseline participant characteristics.

Baseline characteristic	Overall, *N* = 238^a^	Dual‐ART (DRV/r + RGV), *N* = 119^a^	Triple‐ART (DRV/r + TDF/FTC), *N* = 119^a^
Male gender	208 (87%)	105 (88%)	103 (87%)
Age (years)	38 (31, 46)	35 (31, 44)	40 (31, 48)
Ethnic group			
Asian	7 (2.9%)	5 (4.2%)	2 (1.7%)
Black	29 (12%)	16 (13%)	13 (11%)
White	198 (83%)	96 (81%)	102 (86%)
Other	4 (1.7%)	2 (1.7%)	2 (1.7%)
Education (years)	13.0 (10.0, 16.0)	13.0 (10.0, 16.5)	13.0 (9.5, 15.0)
Unknown	46	31	15
Smoking status			
Never	127 (53%)	72 (61%)	55 (46%)
Ex‐smoker	22 (9.2%)	8 (6.7%)	14 (12%)
Current smoker	89 (37%)	39 (33%)	50 (42%)
Log10 plasma HIV‐1 RNA concentration (copies/mL)	4.73 (4.23, 5.11)	4.72 (4.20, 5.19)	4.73 (4.26, 5.00)
CD4 count (cells/mm^3^)	350 (285, 412)	350 (280, 411)	350 (288, 415)
<50	8 (3.4%)	2 (1.7%)	6 (5.0%)
50–199	18 (7.6%)	9 (7.6%)	9 (7.6%)
200–349	92 (39%)	48 (40%)	44 (37%)
350–499	107 (45%)	51 (43%)	56 (47%)
≥500	13 (5.5%)	9 (7.6%)	4 (3.4%)
Nadir CD4 count (cells/mm^3^)	336 (261, 388)	331 (243, 383)	342 (267, 398)
CD4:CD8 ratio	0.37 (0.26, 0.53)	0.38 (0.25, 0.56)	0.36 (0.26, 0.53)
Unknown	12	6	6
HIV CDC clinical stage			
A	204 (86%)	102 (86%)	102 (86%)
B	26 (11%)	14 (12%)	12 (10%)
C	8 (3.4%)	3 (2.5%)	5 (4.2%)
NPZ score (week 0)	0.07 (−0.36, 0.51)	0.03 (−0.34, 0.57)	0.09 (−0.36, 0.46)
Unknown	52	35	17

Abbreviations: ART, antiretroviral therapy; CDC, U.S. Centers for disease control and prevention; DRV/r, darunavir/ritonavir; FTC, Emtricitabine; NPZ, composite cognitive *z*‐score of seven neuropsychological tests; RGV, raltegravir; TDF, tenofovir disoproxil.

^a^ *n* (%); Median (Q1, Q3).

### Baseline biomarker concentrations and associated factors

Plasma biomarker concentrations at W0 are displayed in Table 2. Factors associated with higher biomarker concentrations at W0 included a CD4 count below 50 cells/mm^3^ (NfL, GFAP and CXCL10; *p* < 0.03), higher log_10_ HIV RNA (ratio of GM for the effect of a 1‐unit increase in log_10_ biomarker value: sCD14 1.09, neopterin 1.35, CXCL10 1.50, IL‐6 1.24; *p* < 0.02), increasing age (effect of 10 years of age: NfL 1.27, GFAP 1.11, CXCL10 1.11 and neopterin 1.13; *p* < 0.01) and longer duration of HIV (effect of 5 years: sCD14 1.06 *p* = 0.044) but no other baseline characteristics (Table S3). Age‐adjusted plasma NfL concentrations were greater than population normative concentrations in 32 (13% [95% confidence interval, CI 9.5%, 19%]) individuals. Pairwise rank biomarker correlations at baseline are presented in Figure S2. Moderate‐strength positive correlations were found between neopterin and CXCL10 (Spearman's *ρ* = 0.61); neopterin and sCD14 (*ρ* = 0.41); neopterin and HIV RNA (*ρ* = 0.44); CXCL10 and IL‐6 (*ρ* = 0.40); CXCL10 and HIV RNA (*ρ* = 0.41).

**TABLE 2:** Relative change in biomarker concentrations after 96 weeks on antiretroviral therapy (ART).

Biomarker	Week 0		Week 96		Relative change (%)^b^	95% CI^b^	*p*‐Value^b^
	Median (IQR)^a^	*N*	Median (IQR)^a^	*N*			
NfL (pg/mL)	6.4 (4.6, 8.8)	238	6.1 (4.7, 8.2)	238	−4.4	−9.1, +0.5	0.075
GFAP (pg/mL)	67.9 (54.0, 95.9)	238	62.1 (49.7, 77.8)	238	−14.4	−18.3, −10.3	<0.001
sCD14 (ng/mL)	1405.9 (1182.7, 1655.6)	238	1291.9 (1081.6, 1555.3)	238	−6.8	−9.6, −4.0	<0.001
Neopterin (nmol/L)	14.0 (10.4, 18.5)	238	7.5 (5.7, 9.5)	238	−47.4	−50.1, −44.5	<0.001
CXCL10 (pg/mL)	2074.0 (1350.5, 2954.4)	238	804.6 (615.0, 1080.5)	238	−58.8	−61.6, −55.7	<0.001
IL‐6 (pg/mL)	2.2 (1.4, 3.1)	237	1.4 (0.9, 2.3)	238	−29.5	−36.1, −22.4	<0.001

Abbreviations: CI, confidence interval; CXCL10, C‐X‐C motif chemokine 10; GFAP, glial fibrillary acidic protein; IQR, interquartile range; IL‐6, interleukin 6; NfL, neurofilament light; sCD14, soluble CD14.

^a^ Median (Q1, Q3).

^b^ Paired *t*‐test.

### Change in biomarker concentrations and associated factors

**FIGURE 1:**
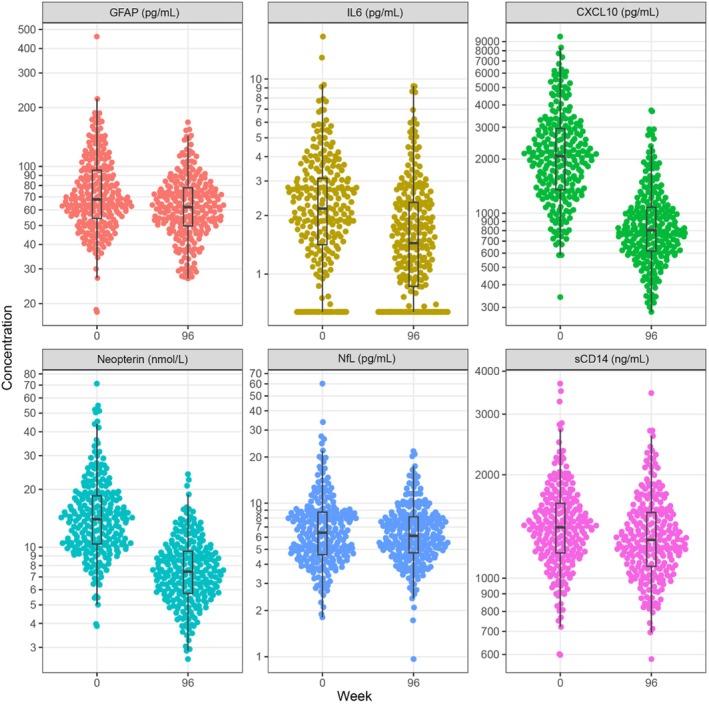
Plasma biomarker concentrations at weeks 0 and 96. From the top left of the figure, concentrations of plasma glial fibrillary acidic protein (GFAP), interleukin 6 (IL‐6), C‐X‐C motif chemokine 10 (CXCL10), neopterin, neurofilament light (NfL) and soluble CD14 (sCD14) at weeks 0 and 96. Median and interquartile range shown as box and whisker plots. Statistically significant decline in GFAP, sCD14, neopterin, CXCL10 and IL‐6 (all *p* < 0.001), with the greatest relative change observed in neopterin (−47.4%) and CXCL10 (−58.8%).

**FIGURE 2:**
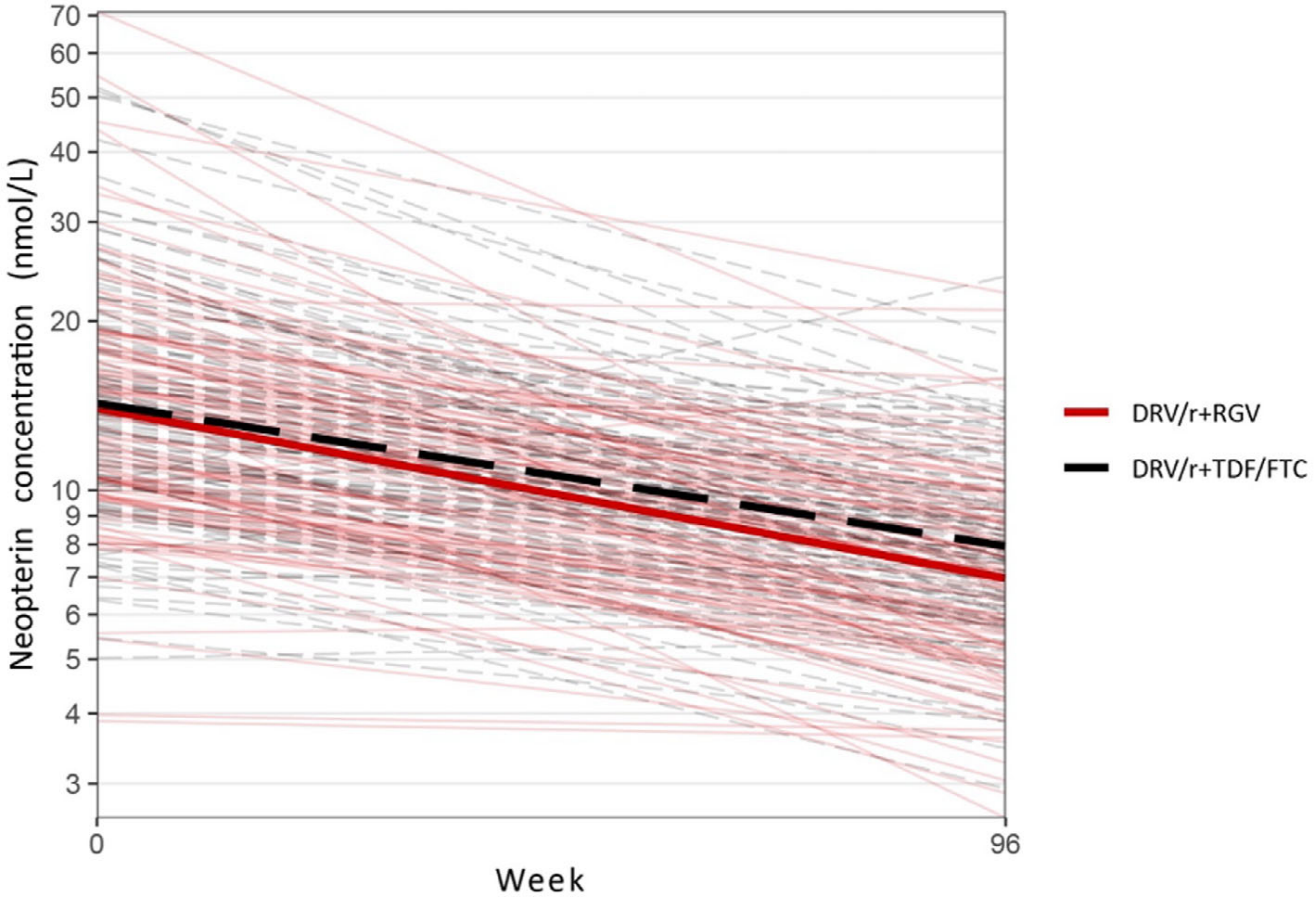
Change in neopterin concentrations over 96 weeks by randomized drug arm. Median change in neopterin concentrations between weeks 0 and 96 by randomized drug arm are displayed as a solid red line (dual‐ART) and dashed black line (triple‐ART). Lower neopterin concentrations were observed at week 96 in the dual‐ versus triple‐ART arm. DRV/r + RGV (darunavir/ritonavir, raltegravir); DRV/r + TDF/FTC (darunavir/ritonavir, tenofovir disoproxil/emtricitabine).

At W96, 222/237 (94%) individuals had plasma HIV RNA concentrations <50 copies/mL (96% triple‐ART, 91% dual‐ART, *p* = 0.06) and NPZ score increased by a mean 0.21 points; 0.21 triple‐ART versus 0.27 dual‐ART (*p* = 0.46). Mean biomarker concentrations decreased from W0 to W96 (RC GFAP −14.4% [95% CI −18.3%, −10.3%] pg/mL, sCD14 −6.8% [95% CI −9.6%, −4.0%] ng/mL, neopterin −47.4% [95% CI −50.1%, −44.5%] nmol/L, CXCL10 −58.8% [95% CI −61.6%, −55.7%] pg/mL, IL‐6 −29.5% [95% CI −36.1%, −22.4%], pg/mL [all *p* < 0.001]; Table 2 and Figure 1, Table S4). However, no statistically significant decline in NfL concentrations was observed (RC NfL −4.4% [95% CI −9.1%, +0.5%], *p* = 0.075; Table 2). The primary outcome measures were the difference in GFAP and NfL concentrations from baseline, with all other outcomes considered as exploratory. At W96, 20/238 (8% 95% CI 5.3%, 13%) individuals had plasma NfL concentrations above population normative means for age, of which 9 had within reference range concentrations at W0 and 11 had persistently elevated concentrations from W0. Elevated age‐adjusted plasma NfL at W96 was associated with elevated W0 concentrations, with no associations observed by W0 CD4 count, plasma HIV RNA concentrations or NPZ score (Tables S5 and S6). No significant association was found between plasma biomarker change scores and age, sex and change in CD8 count; or change in NPZ score (Table 3). However, change in biomarker concentrations was inversely associated with change in CD4: A 20% increase in CD4 was associated with an average reduction in NfL by 5% (95% CI 3%, 7%), GFAP by 3% (95% CI 1%, 5%), IL‐6 by 2% (95% CI −3%, 6%), CXCL10 by 6% (95% CI 3%, 9%), sCD14 by 3% (95% CI 1%, 4%) and neopterin by 4% (95% CI 2%, 7%). Each of those effects was statistically significant (*p* ≤ 0.002), except for IL‐6 (*p* = 0.464). Mean change scores were also compared across trial arms (Table 4). A greater decline in neopterin concentration was observed in those randomized to dual‐ART (RC −50.2% [95% CI −54.0, −46.1]), when compared to triple‐ART (RC −44.3% [95% CI −48.1, −40.3]) (difference in RC 13.1% [95% CI 1.8%, 25.6%], *p* = 0.022) (Figure 2).

**TABLE 3:** Multivariate linear regression analysis of factors associated with change in log_10_ biomarker concentrations between week 0 and week 96.

Characteristic	NfL (log_10_ pg/mL)			GFAP (log_10_ pg/mL)			IL‐6 (log_10_ pg/mL)			CXCL10 (log_10_ pg/mL)			sCD14 (log_10_ ng/mL)			Neopterin (log_10_ nmol/L)		
	10^β^	95% CI^a^	*p*‐Value	10^β^	95% CI^a^	*p*‐Value	10^β^	95% CI^a^	*p*‐Value	10^β^	95% CI^a^	*p*‐Value	10^β^	95% CI^a^	*p*‐Value	10^β^	95% CI^a^	*p*‐Value
(Age‐40)/10 (years)	1.00	0.96, 1.05	0.959	0.98	0.93, 1.03	0.385	0.93	0.83, 1.04	0.203	0.93	0.86, 1.01	0.075	0.97	0.93, 1.00	**0.044**	0.95	0.90, 1.01	0.111
Male	0.96	0.81, 1.13	0.630	0.96	0.80, 1.17	0.707	0.85	0.57, 1.26	0.405	1.30	0.98, 1.73	0.066	0.92	0.82, 1.04	0.176	1.12	0.91, 1.38	0.281
Change in CD4 (log_10_ cells/mm^3^)	0.53	0.42, 0.67	**<0.001**	0.65	0.50, 0.85	**0.002**	0.82	0.47, 1.41	0.464	0.43	0.29, 0.64	**<0.001**	0.70	0.60, 0.83	**<0.001**	0.56	0.42, 0.75	**<0.001**
Change in CD8 (log_10_ cells/mm^3^)	0.86	0.70, 1.05	0.139	0.88	0.70, 1.11	0.287	1.13	0.69, 1.84	0.627	1.13	0.80, 1.60	0.484	1.10	0.95, 1.27	0.203	1.25	0.97, 1.61	0.080
Change in NPZ score	1.05	0.89, 1.23	0.579	1.04	0.86, 1.25	0.700	1.02	0.69, 1.51	0.906	0.91	0.69, 1.21	0.515	1.02	0.90, 1.14	0.792	1.01	0.82, 1.24	0.922

Abbreviations: CXCL10, C‐X‐C motif chemokine 10; GFAP, glial fibrillary acidic protein; IL‐6, Interleukin‐6; NfL, neurofilament light; NPZ, composite cognitive *z*‐score of seven neuropsychological tests; sCD14, Soluble CD14.

^a^ Confidence interval (CI) of 10^β^ (relative change ratio per respective unit change in biomarker). *p*‐Values highlighted in bold indicate factors significantly associated with baseline log_10_ biomarker concentrations.

**TABLE 4:** Relative change in biomarker concentrations between week 0 and week 96 by randomized drug arm.

Biomarker	Dual‐ART (DRV/r + RAL), *N* = 119			Triple‐ART (DRV/r + TDF/FTC), *N* = 119			Difference in relative change^b^	95% CI^b^, ^c^	*p*‐Value^c^
	Median relative change (IQR)^a^	Mean relative change (%)	95% CI^b^	Median relative change (IQR)^a^	Mean relative change (%)	95% CI^a^			
NfL (pg/mL)	+0.7 (−16.1, +20.5)	−3.7	−10.6, +3.8	−0.7 (−18.2, +21.1)	−5.1	−11.3, +1.4	−1.4	−10.9, 9.0	0.779
GFAP (pg/mL)	−13.8 (−30.5, +5.4)	−15.2	−21.2, −8.8	−11.0 (−27.0, +5.8)	−13.6	−18.7, −8.3	2.4	−6.8, 12.5	0.623
sCD14 (ng/mL)	−8.6 (−19.6, +6.9)	−7.5	−11.6, −3.3	−7.6 (−18.7, +7.5)	−6.1	−9.9, −2.2	2.0	−4.0, 8.3	0.526
Neopterin (nmol/L)	−48.2 (−60.5, −34.6)	−50.2	−54.0, −46.1	−44.6 (−57.7, −30.6)	−44.3	−48.1, −40.3	13.1	1.8, 25.6	0.022
CXCL10 (pg/mL)	−58.7 (−71.2, −40.2)	−58.3	−62.4, −53.8	−57.7 (−69.8, −44.4)	−59.2	−63.1, −54.9	−0.8	−14.0, 14.3	0.907
IL‐6 (pg/mL)	−31.3 (−60.2, +11.2)	−30.5	−39.8, −19.8	−21.6 (−53.2, +1.3)	−28.6	−37.4, −18.5	3.5	−14.9, 25.8	0.729

*Note*: Mean log_10_ change scores were back‐transformed into relative change percentages.

Abbreviations: ART, antiretroviral therapy; CI, confidence interval; CXCL10, C‐X‐C motif chemokine 10; DRV/r, darunavir/ritonavir; FTC, emtricitabine; GFAP, glial fibrillary acidic protein; IQR, interquartile range; IL‐6, interleukin‐6; NfL, neurofilament light; sCD14, soluble CD14; RAL, raltegravir; TDF, tenofovir disoproxil.

^a^ Median (Q1, Q3).

^b^ CI for the mean.

^c^ Analysis of covariance (ANCOVA).

## DISCUSSION

We have assessed the effects of dual‐ and triple‐ART on plasma biomarkers of immune activation, neuronal inflammation and injury in treatment naïve persons with HIV commencing ART, and their associations with cognitive function. Over the 96‐week study period, we observed a decline in concentrations of the select plasma biomarkers, including NfL, GFAP, sCD14, neopterin, CXCL10 and IL‐6, which were predominantly associated with improvements in immune function parameters. These findings suggest that commencing ART improved HIV‐associated immune activation and inflammation; findings which are supported by the literature [22, 23]. While global cognitive performance improved over 96 weeks after commencing ART [1], no association was observed with change in biomarker concentrations, and the clinical significance of change in biomarker concentrations therefore remains unclear.

Despite ART, chronic immune activation and inflammation may persist in persons with HIV, increasing the risk of non‐AIDS‐related comorbidities, such as cardiovascular disease and cognitive disorders. Several mechanisms have been suggested as potential contributors, such as viral persistence and transcription in HIV reservoir cells, low‐level HIV RNA replication, gut barrier dysfunction and the presence of co‐infections [24, 25, 26]. The impact of different ART regimens on markers of inflammation remains relatively unexplored. We observed comparable changes in biomarker concentrations between randomized drug arms across the study period, with a greater decline in neopterin favouring dual‐ART; findings which we hypothesize may, in part, be related to reduced neurotoxicity with dual‐ART compared to triple‐ART. However, we acknowledge that this study was not powered to address the effect of drug arm on biomarker concentrations and these observations should be interpreted with caution. Consistent with our findings, Tan et al. reported no differences in changes in plasma biomarkers of inflammation (hsCRP, IL‐6, MCP‐1, TNF d‐dimer and sCD14) over 48 weeks in those initiating ART for the first time with dual‐ART (lopinavir/ritonavir and lamivudine), when compared to triple‐ART (lopinavir/ritonavir and 2NRTIs) [27]. Several other studies have assessed biomarker changes between dual‐ and triple‐ART; however, these are predominantly in the context of virologically suppressed individuals switching ART and are assessing dual‐ART regimens that include a second‐generation INSTI, which differs from our study. A Spanish cohort study in virologically suppressed individuals switching to several different dual‐ART regimens reported an increase in concentrations of IL‐6, d‐dimer and sCD14, when compared to those who continued triple‐ART [28]. While this is an interesting observation, this study is limited by the cohort nature of the analysis and could be confounded by the clinical indications for switch to dual‐ART regimens. In contrast, recent studies of second‐generation INSTI‐based ART have demonstrated either no differences or a reduction in inflammatory biomarker profiles in those switched to dual‐ART [29, 30]. A limitation of our analysis is the dual‐ART regimen we have assessed. Darunavir/ritonavir with raltegravir is not a preferred ART regimen in most ART treatment guidelines and biomarker dynamics may differ in dual‐ART regimens containing second‐generation INSTIs, when compared with the regimen we have assessed.

We observed 32 (13%) individuals to have plasma NfL concentrations above expected populations normative means at baseline and notably this was in the absence of overt neurological symptoms. Consistent with previous literature, factors associated with higher NfL concentrations at baseline were increased age and lower CD4 count [31, 32]. NfL declined longitudinally, with a RC −4.4% from baseline, and only 20 (8%) had persistent plasma NfL concentrations above population‐defined reference thresholds at W96, suggesting that raised plasma NfL concentrations above population thresholds persist in only a small number of individuals after 96 weeks of ART. In contrast, recent data from Calcagno et al., demonstrated raised serum NfL concentrations in 49% of treatment naïve individuals with primary HIV and in 28% after 48 weeks of ART [33]. There may be several reasons why we observed differences in the frequency of individuals with persistently elevated plasma NfL concentrations compared to Calcagno et al. Firstly, previous literature has described elevated NfL concentrations in those with advanced untreated HIV, typically CD4 counts <100 cells/mm^3^, in those with HIV‐associated dementia and in some individuals with primary HIV [32, 34]. Despite the relatively low median CD4 counts at baseline (350 cells/mm^3^) in our study, most had a CD4 count above 200 cells/mm^3^ (84%) and therefore did not have the risk factor for elevated NfL concentration of a CD4 count below 100 cells/mm^3^. Interestingly, we have observed median plasma NfL concentrations across both timepoints to be similar to previously reported values in persons without HIV (median W0 6.4 pg/mL and W96 6.1 pg/mL vs. 9.9 pg/mL) [35]. Of the 32 individuals with elevated NfL at W0, only 11 had persistently elevated NfL at W96. The 2 years of follow‐up on ART in our study may have allowed normalization of plasma concentrations in most individuals.

Strengths of our study include the large sample size and follow‐up over 96 weeks, which allowed assessment of biomarker concentrations and their associations with clinical outcomes for nearly 2 years after ART initiation. The study design also allowed us to assess biomarker concentrations according to ART regimen, providing further insight into the longitudinal effects of ART on markers of inflammation. We were also able to include a control inflammatory marker (IL‐6) as a comparison for the inflammatory markers we considered to be specific to neuronal injury. Despite this, our study has some limitations. Firstly, despite a relatively large sample size for the measurement of biomarker concentrations, longitudinal cognitive assessment was not available for all participants, which limited our ability to perform subgroup analyses based on change in cognitive function. While we observed improved cognitive performance at 96 weeks, with no significant difference between study arms, practice effects are observed in all longitudinal cognitive studies and will be present in this study. However, practice effects are likely limited due to the 96‐week interval between cognitive assessments. The study population was comprised of predominantly white men, and other groups such as women and those of non‐white ethnicity were under‐represented, limiting the generalizability of these findings. All the participants in this study commenced ART at baseline and were followed up over 96 weeks. However, as biomarker concentrations were measured at only two time points (W0 and W96), further details on the temporal dynamics of biomarker changes could not be assessed. The lack of corresponding cerebrospinal fluid samples meant we were unable to determine correlations between plasma and cerebrospinal fluid biomarkers over the study period. We were not able to control for potential confounders such as lifestyle factors, comorbidities or ART‐related metabolic effects, which could have influenced biomarker concentrations. Lastly, as previously described, the ART regimen assessed in this study is not preferred in most ART treatment guidelines, limiting generalizability with other contemporary dual‐ART regimens.

## CONCLUSIONS

Plasma biomarkers of brain health improved following 2 years of ART. Changes in biomarker concentrations were associated predominantly with improvements in CD4 count and partly by randomized drug arm, with a greater decline in neopterin concentration observed over 96 weeks in those randomized to dual‐ART, when compared to those on triple‐ART. Change in cognitive function was not associated with change in biomarker concentrations. Further research in larger scale clinical trials using contemporary dual‐ART regimens is warranted.

## AUTHOR CONTRIBUTIONS

All authors reviewed and approved the final manuscript. **Merle Henderson**: Conceptualisation; funding acquisition; methodology; investigation; formal analysis; writing – original draft preparation; review and editing. **Peter Dutey‐Magni**: Software; formal analysis; writing – review and editing. **Carolina Herrera**: Methodology; writing – review and editing. **Wolfgang Stöhr**: Writing – review and editing. **Alejandro Arenas‐Pinto**: Writing – review and editing. **Owen Swann**: Investigation; writing – review and editing. **Amanda Heslegrave**: Writing – review and editing. **Henrik Zetterberg**: Writing – review and editing; **John Tregoning**: Supervision; writing – review and editing. **Sarah Fidler**: Writing – review and editing. **François Raffi**: Writing – review and editing. **Andrea Calcagno**: Writing – review and editing. **Ab Babiker**: Writing – review and editing. **Alan Winston**: Conceptualisation; funding acquisition; formal analysis; supervision; writing – original draft preparation; review and editing.

## FUNDING INFORMATION

Merle Henderson received a NEAT‐ID Integration Grant Award to complete this work. The biomarker measurements at UCL were supported by the National Institute for Health and Care Research University College London Hospitals Biomedical Research Centre, and the UK Dementia Research Institute at UCL (UKDRI‐1003).

## CONFLICT OF INTEREST STATEMENT

Merle Henderson has received support to attend scientific conferences from ViiV and Gilead Sciences and has received research grant funding from NEAT‐ID. Henrik Zetterberg has served at scientific advisory boards and/or as a consultant for Abbvie, Acumen, Alector, Alzinova, ALZpath, Amylyx, Annexon, Apellis, Artery Therapeutics, AZTherapies, Cognito Therapeutics, CogRx, Denali, Eisai, Enigma, LabCorp, Merry Life, Nervgen, Novo Nordisk, Optoceutics, Passage Bio, Pinteon Therapeutics, Prothena, Quanterix, Red Abbey Labs, reMYND, Roche, Samumed, Siemens Healthineers, Triplet Therapeutics and Wave, has given lectures sponsored by Alzecure, BioArctic, Biogen, Cellectricon, Fujirebio, Lilly, Novo Nordisk, Roche and WebMD, and is a co‐founder of Brain Biomarker Solutions in Gothenburg AB (BBS), which is a part of the GU Ventures Incubator Program (outside submitted work). Amanda Heslegrave has served as a consultant for Quanterix.

## Supporting information


**Table S1.** Baseline characteristics of those in the main trial, when compared to those in the biomarker cohort.
**Table S2.** Sensitivity analysis for missingness of NPZ scores across the biomarker cohort.
**Table S3.** Multivariate linear regression analysis of factors associated with log_10_ biomarker concentrations at week 0.
**Table S4.** Absolute change in biomarker concentrations after 96 weeks on ART.
**Table S5.** Characteristics of participants with elevated NfL concentrations at week 96.
**Table S6.** Multivariate logistic regression analysis of factors associated with elevated NfL concentrations at week 96.
**Figure S1.** CONSORT diagram of NEAT biomarker study.
**Figure S2.** Pairwise rank biomarker correlation matrix at week 0.

## Data Availability

The data that support the findings of this study are available on request from the corresponding author. The data are not publicly available due to privacy or ethical restrictions.
