# Supplementary material for: Longitudinal changes in plasma biomarkers of immune activation, neuronal inflammation and injury in persons with HIV initiating ART
Source: HIV Med. 2026 Apr 16;27(7):1132–43. doi: 10.1111/hiv.70236 (PMC13340942; doi:10.1111/hiv.70236)
Supplement: Supplementary file 1 — Table S1. Baseline characteristics of those in the main trial, when compared to those in the biomarker cohort. Table S2. Sensitivity analysis for missingness of NPZ scores across the biomarker cohort. Table S3. Multivariate linear regression analysis of factors associated with log10 biomarker concentrations at week 0. Table S4. Absolute change in biomarker concentrations after 96 weeks on ART. Table S5. Characteristics of participants with elevated NfL concentrations at week 96. Table S6. Multivariate logistic regression analysis of factors associated with elevated NfL concentrations at week 96. Figure S1. CONSORT diagram of NEAT biomarker study. Figure S2. Pairwise rank biomarker correlation matrix at week 0. [file HIV-27-1132-s001.docx]

**Supplementary materials**

**S1 Supplementary tables**

| **Supplementary table 1. Baseline characteristics of those in the main trial, when compared to those in the biomarker cohort.** | | | | |
| --- | --- | --- | --- | --- |
| **Baseline characteristic** | **Biomarker cohort**  N = 238*^1^* | **Not in biomarker cohort**  N = 567*^1^* | **p-value***^2^* | **Overall**  N = 805*^1^* |
| Trial arm |  |  | 0.945 |  |
| Dual-ART (DRV/r + RGV) | 119 (50%) | 282 (50%) |  | 401 (50%) |
| Triple-ART (DRV/r + TDF/FTC) | 119 (50%) | 285 (50%) |  | 404 (50%) |
| Gender |  |  | 0.647 |  |
| Male | 208 (87%) | 502 (89%) |  | 710 (88%) |
| Female | 30 (13%) | 65 (11%) |  | 95 (12%) |
| Age (years) | 38 (31, 46) | 38 (31, 46) | 0.702 | 38 (31, 46) |
| Age group (years) |  |  | 0.468 |  |
| <35 | 98 (41%) | 227 (40%) |  | 325 (40%) |
| 35 - <45 | 69 (29%) | 188 (33%) |  | 257 (32%) |
| ≥45 | 71 (30%) | 152 (27%) |  | 223 (28%) |
| Ethnic group |  |  | 0.330 |  |
| Asian | 7 (2.9%) | 12 (2.1%) |  | 19 (2.4%) |
| Black | 29 (12%) | 72 (13%) |  | 101 (13%) |
| White | 198 (83%) | 460 (81%) |  | 658 (82%) |
| Other | 4 (1.7%) | 23 (4.1%) |  | 27 (3.4%) |
| Education (years) | 13.0 (10.0, 16.0) | 13.0 (11.0, 17.0) | 0.166 | 13.0 (10.0, 16.0) |
| (Missing) | 46 | 457 |  | 503 |
| Smoking status |  |  | 0.202 |  |
| Never | 127 (53%) | 262 (47%) |  | 389 (49%) |
| Ex-smoker | 22 (9.2%) | 66 (12%) |  | 88 (11%) |
| Current smoker | 89 (37%) | 233 (42%) |  | 322 (40%) |
| (Missing) | 0 | 6 |  | 6 |
| HIV-1 RNA concentration (copies/mL) at week 0 | 53,286 (17,113, 127,381) | 61,500 (21,870, 150,001) | 0.079 | 59,094 (20,250, 142,430) |
| log10 HIV-1 RNA concentration (copies/mL) at week 0 | 4.73 (4.23, 5.11) | 4.79 (4.34, 5.18) | 0.079 | 4.77 (4.31, 5.15) |
| CD4 cell count (cells/mm^3^) at week 0 | 350 (285, 412) | 329 (244, 393) | 0.009 | 333 (253, 399) |
| CD4 cell count (cells/mm^3^) at week 0 |  |  | 0.048 |  |
| <50 | 8 (3.4%) | 21 (3.7%) |  | 29 (3.6%) |
| 50 - 199 | 18 (7.6%) | 76 (13%) |  | 94 (12%) |
| 200 - 349 | 92 (39%) | 241 (43%) |  | 333 (41%) |
| 350 - 499 | 107 (45%) | 201 (35%) |  | 308 (38%) |
| ≥500 | 13 (5.5%) | 28 (4.9%) |  | 41 (5.1%) |
| CD4 cell count nadir (cells/mm^3^) | 336 (261, 388) | 303 (232, 367) | <0.001 | 311 (242, 374) |
| (Missing) | 0 | 1 |  | 1 |
| CD4:CD8 ratio | 0.37 (0.26, 0.53) | 0.36 (0.24, 0.51) | 0.201 | 0.37 (0.25, 0.52) |
| (Missing) | 12 | 20 |  | 32 |
| HIV CDC clinical stage |  |  | 0.302 |  |
| A | 204 (86%) | 462 (81%) |  | 666 (83%) |
| B | 26 (11%) | 75 (13%) |  | 101 (13%) |
| C | 8 (3.4%) | 30 (5.3%) |  | 38 (4.7%) |
| Ever CNS AIDS event | 0 (0%) | 0 (0%) | >0.999 | 0 (0%) |
| (Missing) | 44 | 418 |  | 462 |
| Diabetes at baseline | 1 (0.5%) | 2 (1.3%) | 0.582 | 3 (0.9%) |
| (Missing) | 44 | 418 |  | 462 |
| CNS-related disorder at screening | 10 (5.2%) | 8 (5.4%) | 0.930 | 18 (5.2%) |
| (Missing) | 44 | 418 |  | 462 |
| NPZ score (week 0) | 0.07 (-0.36, 0.51) | -0.01 (-0.35, 0.31) | 0.165 | 0.02 (-0.35, 0.44) |
| (Missing) | 52 | 480 |  | 532 |
| *^1^*n (%); Median (Q1, Q3), *^2^*Pearson's Chi-squared test; Wilcoxon rank sum test; Fisher's exact test. Abbreviations: CDC (U.S. Centers for disease control and prevention), CNS (Central nervous system), DRV/r (Darunavir/ritonavir), FTC (Emtricitabine), NPZ (composite cognitive z-score of 7 neuropsychological tests), RGV (Raltegravir), RNA (Ribonucleic acid), TDF (Tenofovir disoproxil). | | | | |
|  | | | | |

| **Supplementary table 2. Sensitivity analysis for missingness of NPZ scores across the biomarker cohort.** | | | | |
| --- | --- | --- | --- | --- |
| **Characteristic** | **NPZ missing** N = 74*^1^* | **NPZ not missing** N = 164*^1^* | **p-value***^2^* | **Overall** N = 238*^1^* |
| Trial arm |  |  | 0.002 |  |
| Dual-ART (DRV/r + RGV) | 48 (40%) | 71 (60%) |  | 119 (100%) |
| Triple-ART (DRV/r + TDF/FTC) | 26 (22%) | 93 (78%) |  | 119 (100%) |
| Gender |  |  | 0.049 |  |
| Male | 60 (29%) | 148 (71%) |  | 208 (100%) |
| Female | 14 (47%) | 16 (53%) |  | 30 (100%) |
| Age (years) | 37 (31, 43) | 39 (31, 47) | 0.431 | 38 (31, 46) |
| Age group (years) |  |  | 0.296 |  |
| <35 | 33 (34%) | 65 (66%) |  | 98 (100%) |
| 35 - <45 | 24 (35%) | 45 (65%) |  | 69 (100%) |
| ≥45 | 17 (24%) | 54 (76%) |  | 71 (100%) |
| Ethnic group |  |  | 0.627 |  |
| Asian | 2 (29%) | 5 (71%) |  | 7 (100%) |
| Black | 11 (38%) | 18 (62%) |  | 29 (100%) |
| White | 59 (30%) | 139 (70%) |  | 198 (100%) |
| Other | 2 (50%) | 2 (50%) |  | 4 (100%) |
| Education (years) | 15.0 (11.0, 17.5) | 13.0 (10.0, 15.0) | 0.066 | 13.0 (10.0, 16.0) |
| (Missing) | 46 | 0 |  | 46 |
| Smoking status |  |  | 0.448 |  |
| Never | 44 (35%) | 83 (65%) |  | 127 (100%) |
| Ex-smoker | 6 (27%) | 16 (73%) |  | 22 (100%) |
| Current smoker | 24 (27%) | 65 (73%) |  | 89 (100%) |
| HIV-1 RNA concentration (copies/mL) at week 0 | 41,525 (14,090, 108,986) | 55,139 (19,396, 133,245) | 0.247 | 53,286 (17,113, 127,381) |
| log10 HIV-1 RNA concentration (copies/mL) at week 0 | 4.62 (4.15, 5.04) | 4.74 (4.29, 5.12) | 0.247 | 4.73 (4.23, 5.11) |
| CD4 cell count (cells/mm^3^) at week 0 | 356 (285, 412) | 349 (284, 411) | 0.693 | 350 (285, 412) |
| CD4 cell count (cells/mm^3^) at week 0 |  |  | 0.875 |  |
| <50 | 1 (13%) | 7 (88%) |  | 8 (100%) |
| 50 - 199 | 5 (28%) | 13 (72%) |  | 18 (100%) |
| 200 - 349 | 29 (32%) | 63 (68%) |  | 92 (100%) |
| 350 - 499 | 35 (33%) | 72 (67%) |  | 107 (100%) |
| ≥500 | 4 (31%) | 9 (69%) |  | 13 (100%) |
| CD4 cell count nadir (cells/mm^3^) | 343 (259, 399) | 327 (262, 380) | 0.275 | 336 (261, 388) |
| CD4:CD8 ratio | 0.36 (0.24, 0.57) | 0.38 (0.28, 0.53) | 0.478 | 0.37 (0.26, 0.53) |
| (Missing) | 3 | 9 |  | 12 |
| HIV CDC clinical stage |  |  | 0.762 |  |
| A | 62 (30%) | 142 (70%) |  | 204 (100%) |
| B | 9 (35%) | 17 (65%) |  | 26 (100%) |
| C | 3 (38%) | 5 (63%) |  | 8 (100%) |
| Ever CNS AIDS event | 0 (NA%) | 0 (NA%) | >0.999 | 0 (NA%) |
| (Missing) | 44 | 0 |  | 44 |
| Diabetes at baseline | 0 (0%) | 1 (100%) | >0.999 | 1 (100%) |
| (Missing) | 44 | 0 |  | 44 |
| CNS-related disorder at screening | 2 (20%) | 8 (80%) | 0.655 | 10 (100%) |
| (Missing) | 44 | 0 |  | 44 |
| NPZ score (week 0) | 0.05 (-0.24, 0.49) | 0.07 (-0.37, 0.52) | 0.766 | 0.07 (-0.36, 0.51) |
| (Missing) | 52 | 0 |  | 52 |
| *^1^*n (%); Median (Q1, Q3), *^2^*Pearson's Chi-squared test; Wilcoxon rank sum test; Fisher's exact test. Abbreviations: CDC (U.S. Centers for disease control and prevention), CNS (Central nervous system), DRV/r (Darunavir/ritonavir), FTC (Emtricitabine), NPZ (composite cognitive z-score of 7 neuropsychological tests), RGV (Raltegravir), RNA (Ribonucleic acid), TDF (Tenofovir disoproxil). | | | | |
|  | | | | |

| **Supplementary table 3. Multivariate linear regression analysis of factors associated with log10 biomarker concentrations at week 0.** | | | | | | | | | | | | | | | | | | |
| --- | --- | --- | --- | --- | --- | --- | --- | --- | --- | --- | --- | --- | --- | --- | --- | --- | --- | --- |
|  | **NfL**  **(log10 pg/mL)** | | | **GFAP**  **(log10 pg/mL)** | | | **IL-6**  **(log10 pg/mL)** | | | **CXCL10**  **(log10 pg/mL)** | | | **sCD14**  **(log10 ng/mL)** | | | **Neopterin**  **(log10 nmol/L)** | | |
| **Baseline characteristic** | **10ᵝ** | **95% CI***^1^* | **p-value** | **10ᵝ** | **95% CI***^1^* | **p-value** | **10ᵝ** | **95% CI***^1^* | **p-value** | **10ᵝ** | **95% CI***^1^* | **p-value** | **10ᵝ** | **95% CI***^1^* | **p-value** | **10ᵝ** | **95% CI***^1^* | **p-value** |
| (Age-40)/10 (years) | 1.27 | 1.19, 1.35 | **<0.001** | 1.11 | 1.05, 1.17 | **<0.001** | 1.02 | 0.92, 1.13 | 0.728 | 1.11 | 1.03, 1.20 | **0.007** | 1.03 | 0.99, 1.07 | 0.187 | 1.13 | 1.06, 1.19 | **<0.001** |
| Male | 1.14 | 0.93, 1.41 | 0.200 | 0.88 | 0.72, 1.06 | 0.176 | 0.99 | 0.70, 1.38 | 0.935 | 0.84 | 0.65, 1.07 | 0.159 | 1.01 | 0.88, 1.15 | 0.921 | 0.93 | 0.77, 1.13 | 0.477 |
| CD4 count (cells/mm^3^) |  |  |  |  |  |  |  |  |  |  |  |  |  |  |  |  |  |  |
| *<50* | — | — |  | — | — |  | — | — |  | — | — |  | — | — |  | — | — |  |
| *50-199* | 0.51 | 0.35, 0.74 | **<0.001** | 0.97 | 0.69, 1.37 | 0.858 | 1.10 | 0.60, 2.01 | 0.765 | 0.65 | 0.42, 1.02 | 0.060 | 1.06 | 0.83, 1.34 | 0.657 | 1.13 | 0.80, 1.60 | 0.474 |
| *200-349* | 0.48 | 0.35, 0.67 | **<0.001** | 0.63 | 0.47, 0.85 | **0.003** | 0.90 | 0.53, 1.53 | 0.697 | 0.64 | 0.43, 0.94 | **0.022** | 0.93 | 0.75, 1.15 | 0.491 | 0.87 | 0.64, 1.17 | 0.358 |
| *350-499* | 0.44 | 0.32, 0.61 | **<0.001** | 0.62 | 0.46, 0.84 | **0.002** | 0.91 | 0.54, 1.54 | 0.720 | 0.64 | 0.44, 0.95 | **0.026** | 0.96 | 0.78, 1.18 | 0.685 | 0.89 | 0.66, 1.20 | 0.429 |
| *≥500* | 0.46 | 0.31, 0.69 | **<0.001** | 0.61 | 0.42, 0.89 | **0.010** | 0.76 | 0.39, 1.47 | 0.415 | 0.57 | 0.35, 0.93 | **0.023** | 1.07 | 0.82, 1.39 | 0.610 | 0.81 | 0.56, 1.18 | 0.270 |
| Log^10^ HIV-1 RNA (copies/mL) | 1.08 | 0.97, 1.20 | 0.176 | 1.04 | 0.94, 1.15 | 0.413 | 1.24 | 1.04, 1.48 | **0.016** | 1.50 | 1.32, 1.70 | **<0.001** | 1.09 | 1.02, 1.17 | **0.016** | 1.35 | 1.22, 1.49 | **<0.001** |
| Education (years) | 1.01 | 0.99, 1.02 | 0.222 | 1.01 | 0.99, 1.02 | 0.475 | 1.00 | 0.98, 1.03 | 0.744 | 1.01 | 1.00, 1.03 | 0.156 | 0.99 | 0.99, 1.00 | 0.272 | 1.00 | 0.98, 1.01 | 0.850 |
| Duration of known HIV (years)^3^ | 1.02 | 0.94, 1.11 | 0.579 | 0.98 | 0.91, 1.06 | 0.697 | 1.09 | 0.95, 1.25 | 0.203 | 1.06 | 0.96, 1.17 | 0.258 | 1.06 | 1.00, 1.12 | **0.044** | 1.03 | 0.96, 1.12 | 0.403 |
| Smoking status |  |  |  |  |  |  |  |  |  |  |  |  |  |  |  |  |  |  |
| *Never* | — | — |  | — | — |  | — | — |  | — | — |  | — | — |  | — | — |  |
| *Ex-smoker* | 0.96 | 0.77, 1.18 | 0.675 | 1.00 | 0.82, 1.22 | 0.993 | 1.06 | 0.75, 1.50 | 0.744 | 0.93 | 0.72, 1.20 | 0.593 | 0.92 | 0.80, 1.06 | 0.230 | 0.96 | 0.78, 1.16 | 0.647 |
| *Current smoker* | 1.08 | 0.95, 1.23 | 0.220 | 0.90 | 0.80, 1.02 | 0.089 | 0.96 | 0.78, 1.19 | 0.697 | 0.97 | 0.83, 1.14 | 0.729 | 1.05 | 0.96, 1.14 | 0.288 | 0.93 | 0.82, 1.05 | 0.220 |
| NPZ score | 1.08 | 0.96, 1.20 | 0.186 | 0.96 | 0.87, 1.06 | 0.431 | 0.96 | 0.81, 1.15 | 0.686 | 1.03 | 0.90, 1.17 | 0.682 | 1.01 | 0.94, 1.09 | 0.698 | 1.07 | 0.97, 1.19 | 0.183 |
| *^1^*CI = Confidence Interval of 10ᵝ (ratio of geometric means per respective unit change in biomarker) ^2^Education in years centred on 13 ^3^Time since first positive HIV serology 1 unit=5 years. P-values highlighted in bold indicate factors significantly associated with baseline log^10^ biomarker concentrations. Abbreviations: CXCL10 (C-X-C motif chemokine 10), GFAP (Glial fibrillary acidic protein), IL-6 (Interleukin 6), NfL (Neurofilament light), NPZ (composite cognitive z-score of 7 neuropsychological tests), RNA (Ribonucleic acid), sCD14 (Soluble CD14). | | | | | | | | | | | | | | | | | | |

| **Supplementary table 4. Absolute change in biomarker concentrations after 96 weeks on ART.** | | | | | | | |
| --- | --- | --- | --- | --- | --- | --- | --- |
|  | **Week 0** | | **Week 96** | |  |  |  |
| **Biomarker** | Median (IQR)*^1^* | N | Median (IQR)*^1^* | N | **Absolute mean difference***^2^* | **95% CI***^2,3^* | **p-value***^2^* |
| log10 NFL (pg/mL) | 0.808 (0.664, 0.942) | 238 | 0.788 (0.677, 0.911) | 238 | -0.02 | -0.04, 0.00 | 0.075 |
| log10 GFAP (pg/mL) | 1.832 (1.733, 1.982) | 238 | 1.793 (1.696, 1.891) | 238 | -0.07 | -0.09, -0.05 | <0.001 |
| log10 IL6 (pg/mL) | 0.336 (0.150, 0.491) | 237 | 0.160 (-0.063, 0.368) | 238 | -0.15 | -0.19, -0.11 | <0.001 |
| log10 CXCL10 (pg/mL) | 3.317 (3.130, 3.470) | 238 | 2.906 (2.789, 3.034) | 238 | -0.38 | -0.42, -0.35 | <0.001 |
| log10 sCD14 (ng/mL) | 3.148 (3.073, 3.219) | 238 | 3.111 (3.034, 3.192) | 238 | -0.03 | -0.04, -0.02 | <0.001 |
| log10 neopterin (nmol/L) | 1.145 (1.016, 1.268) | 238 | 0.872 (0.759, 0.980) | 238 | -0.28 | -0.30, -0.26 | <0.001 |
| *^1^*Median (Quartile 1, Quartile 3), *^2^*Paired t-test, *^3^*CI = Confidence Interval. Abbreviations: ART (Antiretroviral therapy), CXCL10 (C-X-C motif chemokine 10), GFAP (Glial fibrillary acidic protein), IL-6 (Interleukin 6), NfL (Neurofilament light), sCD14 (Soluble CD14). | | | | | | | |

| **Supplementary table 5. Characteristics of participants with elevated NfL concentrations at week 96.** | | | |
| --- | --- | --- | --- |
|  | **W0 NfL status** | |  |
| **Characteristic** | **Normal** N = 9*^1^* | **Elevated** N = 11*^1^* | **Overall** N = 20*^1^* |
| Age (years) | 43 (39, 48) | 40 (35, 48) | 42 (36, 48) |
| CD4 count (cells/mm^3^) at week 0 | 348 (315, 368) | 298 (225, 399) | 316 (279, 384) |
| CD4 count (cells/mm^3^) at week 96 | 649 (564, 709) | 509 (424, 687) | 582 (449, 688) |
| NfL (pg/mL) at week 0 | 8.3 (7.9, 8.5) | 12.0 (10.9, 12.7) | 10.6 (8.3, 12.5) |
| NfL (pg/mL) at week 96 | 11.5 (10.4, 14.0) | 13.5 (11.4, 15.6) | 12.6 (10.9, 15.3) |
| HIV RNA concentration (copies/mL) at week 0 | 56,539 (13,603, 214,290) | 85,133 (15,326, 214,601) | 82,618 (14,465, 214,446) |
| HIV RNA concentration ≥50 copies per mL at week 96 | 1 (11%) | 1 (9.1%) | 2 (10%) |
| *^1^*Median (Q1, Q3); n (%). Abbreviations: RNA (Ribonucleic acid), NfL (neurofilament light chain protein). | | | |

| **Supplementary table 6. Multivariate logistic regression analysis of factors associated with elevated NfL concentrations at Week 96.** | | | |
| --- | --- | --- | --- |
| **Characteristic** | **Estimate** | **Standard error** | **p-value** |
| Age-40/10 (years) | 1.00 | 0.33 | 0.996 |
| Male gender | 0.41 | 0.96 | 0.360 |
| CD4 count (cells/mm^3^) |  |  |  |
| *<50* | 0.00 | 1337. | 0.990 |
| *50-199* | 0.52 | 1.17 | 0.577 |
| *200-349* | 1.00 (ref) | - | - |
| *350-499* | 0.58 | 0.66 | 0.412 |
| *≥500* | 0.58 | 1.28 | 0.674 |
| Log^10^ HIV-1 RNA (copies/mL) | 1.29 | 0.47 | 0.592 |
| NPZ score at W0 | 1.00 | 0.51 | 0.994 |
| Elevated NfL at W0 | 20.20 | 0.64 | <0.0001 |
| *^1^*Median (Q1, Q3); n (%). Abbreviations: RNA (Ribonucleic acid), NfL (neurofilament light chain protein), NPZ (composite cognitive z-score of 7 neuropsychological tests). | | | |

**S2 Supplementary figures**

**
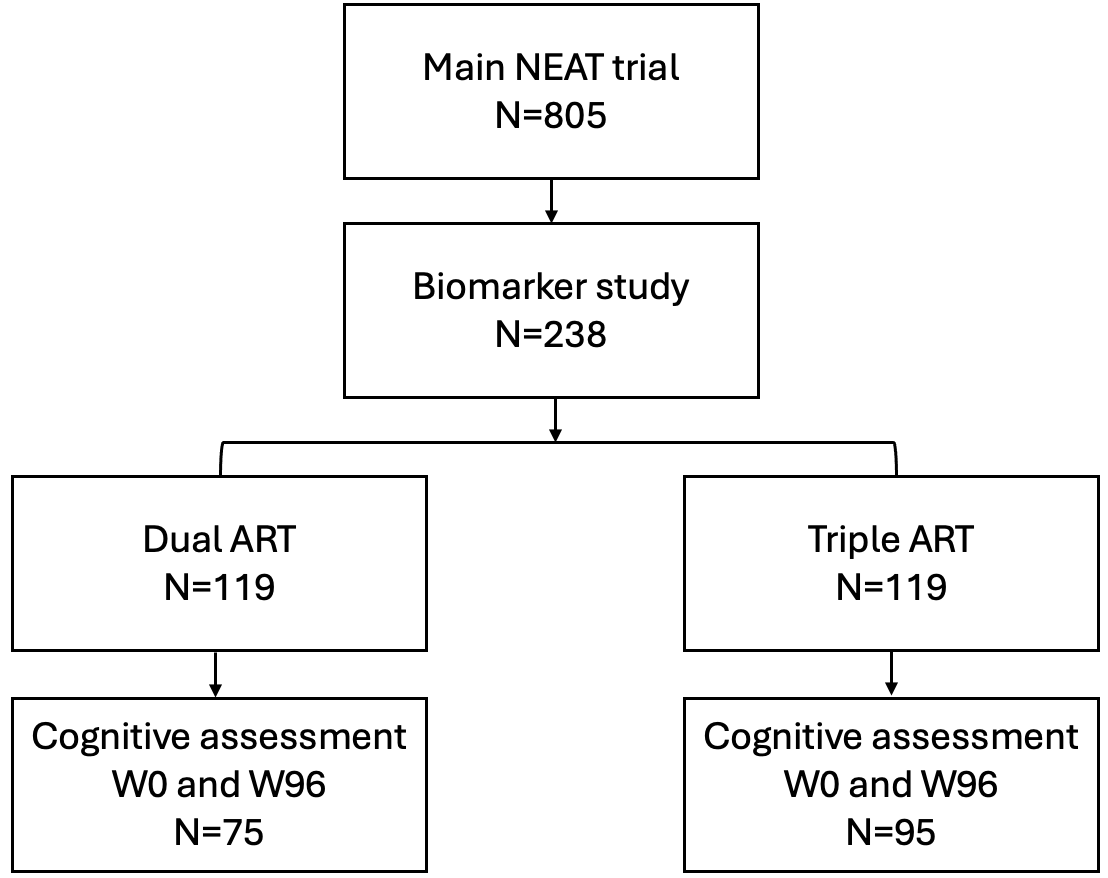
**

**Supplementary figure 1. CONSORT diagram of NEAT biomarker study.**

Abbreviations: ART (antiretroviral therapy), W0 (week 0), W96 (week 96). Figure legend: Of 805 individuals enrolled in the NEAT001/ANRS143 trial, 238 had stored plasma samples at W0 and W96 and were included in this biomarker analysis, of which 170 had corresponding longitudinal cognitive assessments. Of these 238 individuals, 119 were randomized to dual-ART, and 119 to triple-ART; longitudinal cognitive assessment available in 75 and 95, respectively.


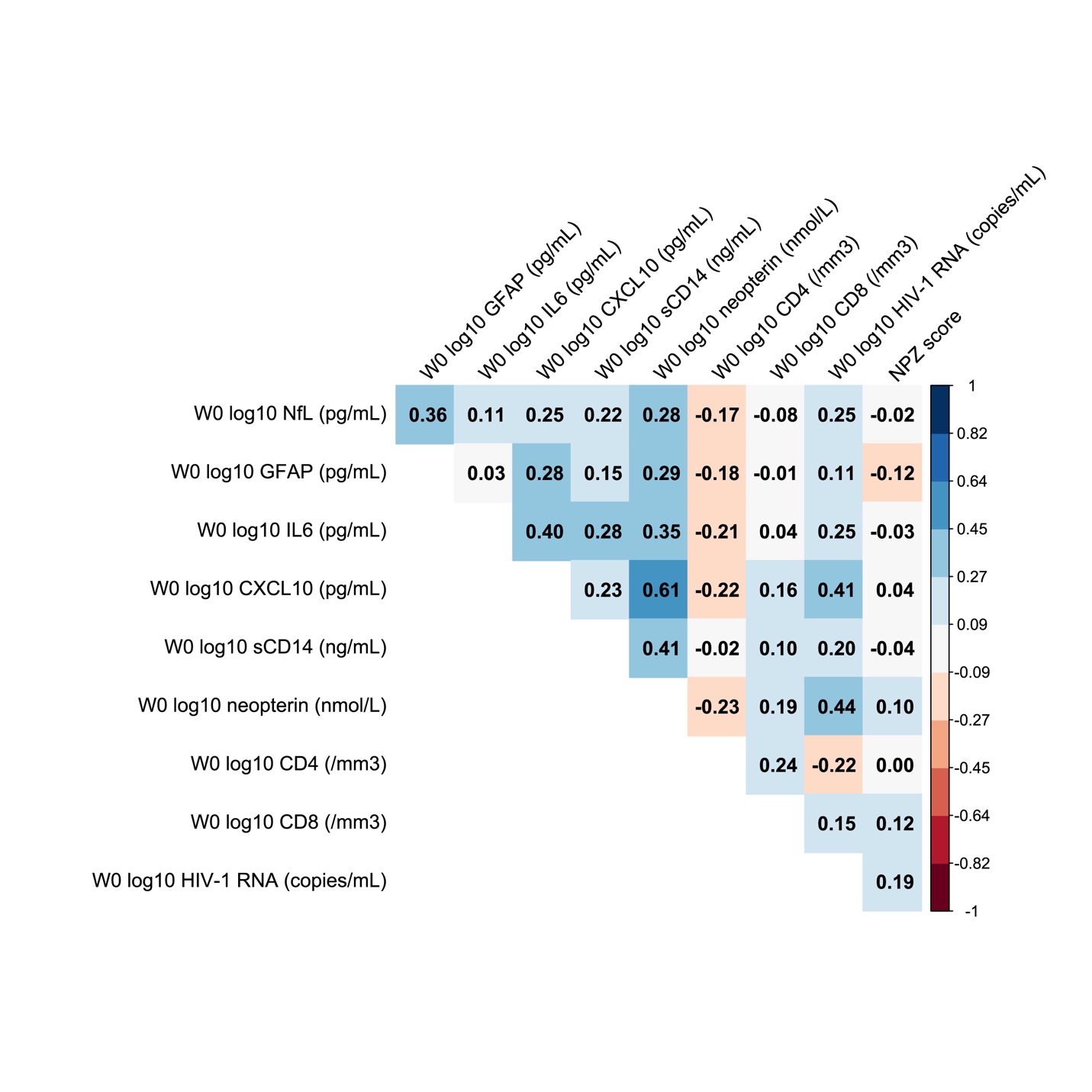


**Supplementary figure 2. Pairwise rank biomarker correlation matrix at week 0.**

Heat map depicting *ρ* values for bivariate correlations between biomarkers at baseline. Moderate-strength positive correlations were found between neopterin and CXCL10 (Spearman’s *ρ*=0.61); neopterin and sCD14 (*ρ*=0.41); neopterin and HIV RNA (*ρ*=0.44); CXCL10 and IL6 (*ρ*=0.40); CXCL10 and HIV RNA (*ρ*=0.41).
